# The Efficacy of Ketogenic Diet in 60 Chinese Patients With Dravet Syndrome

**DOI:** 10.3389/fneur.2019.00625

**Published:** 2019-06-13

**Authors:** Xiaojuan Tian, Jiaoyang Chen, Jing Zhang, Xiaoling Yang, Taoyun Ji, Yao Zhang, Ye Wu, Fang Fang, Xiru Wu, Yuehua Zhang

**Affiliations:** ^1^Department of Pediatrics, Peking University First Hospital, Beijing, China; ^2^Department of Neurology, Beijing Children's Hospital, Capital Medical University, National Center for Children' Health, Beijing, China

**Keywords:** ketogenic diet, Dravet syndrome, pharmaco-resistant, efficacy, safety

## Abstract

**Objective:** To evaluate the efficacy and safety of ketogenic diet (KD) in patients with Dravet syndrome (DS).

**Methods:** 60 DS patients receiving treatment of KD for more than 12 weeks from 2009 to 2018 were analyzed retrospectively. Modified Johns Hopkins protocol was used to initiate KD. Seizure frequency, electroencephalogram (EEG), cognition, language, and motor function of the patients were assessed. Side effects were monitored and adjusted accordingly. SPSS 23.0 software was used for all statistical analysis.

**Results:** In total, 60 DS patients (34 boys, 26 girls) received treatment of KD for more than 12 weeks, and among them 41 (68.3%) patients remained on the diet for more than 24 weeks, 22 (36.7%) patients for more than 48 weeks. Seizures in 35 patients (58.3%) were reduced by over 50% at 12 weeks, and the KD effect was observed within 2 weeks in most of them. At 24 weeks, 61.1% (25/41) of the patients had a >50% seizure reduction. At 48 weeks, 77.3% (17/22) had an over 50% reduction in their seizure frequency. With the treatment of KD in the 60 DS patients, 10 patients had ever been seizure free for 12 months to 24 months (The median duration was 20 months). In 10 KD-effective patients, the background rhythm of their EEG showed obvious improvement, and interictal epileptic discharges decreased significantly. Cognitive function of 22 patients was improved. Language progressed in 14 patients. Motor function was improved in 13 patients. The efficacy of KD in DS patients did not correlate with the seizure onset age, the starting age of KD treatment, the *SCN1A* mutation and the numbers of antiepileptic drugs combined with KD treatment. The main adverse reactions of KD in the treatment process were gastrointestinal symptoms and metabolic disorders.

**Conclusions:** KD treatment in DS patients has many advantages, including working rapidly, being effective in more than half of the DS patients and tolerable adverse reactions. Pharmoco-resistant DS patients are suggested to receive KD treatment.

## Introduction

Dravet syndrome (DS) is a genetic and pharmaco-resistant infantile epilepsy syndrome, characterized by prolonged, febrile and afebrile seizures. Most patients had cognitive impairment, and comorbidities, including autism-like behavior and attention problems. Conventional antiepileptic drugs (AEDs) are not effective enough in most DS patients (1). Newer AEDs are generally reported effective in refractory epilepsy, but all have been reported to have efficacy similar to that of the conventional AEDs. Whether their availability has improved the overall prognosis of epilepsy, therefore, remains controversial (2, 3). Ketogenic diet (KD), a high-fat, adequate-protein and low carbohydrate diet, was first introduced as a therapeutic method for epileptic seizures in 1921 (4). Currently, KD has been widely used in many different countries including China. Recent studies have demonstrated that KD therapy is a valuable feasible alternative to children with intractable epilepsy including DS (5–9). We had a retrospective clinical observation of the effect and safety of KD in DS patients in a Chinese cohort.

## Materials and Methods

### Clinical Data

The DS patients were collected from January 2009 to August 2018 in the Pediatric Department of Peking University First Hospital. The clinical diagnosis of DS included (10): (1) a prolonged unilateral or bilateral clonic or tonic-clonic seizure onset in the first year of life, often triggered by fever (average age of onset 6 months); (2) multiple seizure types (myoclonic, focal, atypical absences) in addition to seizures triggered by fever after 1 year of age; (3) usual occurrence of status epilepticus (SE); (4) normal early development and subsequent delay in psychomotor development, ataxia and pyramidal signs; (5) normal interictal electroencephalography (EEG) in the first year of life followed by generalized, focal, or multifocal discharges; (6) seizures are refractory to antiepileptic drugs.

The inclusion patients should be with a clinical diagnosis of DS. The patients had two or more episodes of seizure every month after regular treatment with at least two AEDs. This was their first attempt to KD therapy and received the KD treatment for 12 weeks or longer at our last follow-up. The study was approved by the Ethics Committee of Peking University First Hospital. Written informed consents were obtained from their parents or legal guardians. Sixty patients were enrolled in this study.

### KD Initiation

Modified Johns Hopkins protocol was used to initiate KD after auxiliary examinations to exclude fat or ketone body metabolism disorders or mitochondrial diseases, as well as other severe systemic diseases (11). The diet has a lipid-to-non-lipid ratio of 4:1. The calories provided to these patients were 75% of daily recommendation for healthy peers. The liquid intake should be less than the physiological requirement. Supplements with potassium citrate, multi-vitamins, essential minerals and calcic agent without sucrose and lactose were provided in daily diet. All 60 patients were admitted inpatients and closely monitored for the internal environment and any acute adverse effect for the first week in the hospital. Parents were educated to prepare the KD at home.

### Effect Observation

Parents were assigned to write diaries which recorded the seizure features, frequency, as well as tolerability and complications associated with KD. After the initiation of the KD therapy, urinary ketone bodies, blood sugar, and blood β-hydroxybutyric acid were measured closely. Patients' weight and height, blood biochemical items including serum lipids, urinary ultrasonic examinations were monitored. Adverse effects were monitored and then adjusted accordingly. Patients were followed up by telephone every month and regular outpatient visits to pediatric neurologists and nutritionists.

The seizure frequency of 3 months before the initiation of KD was recorded as baseline. The seizures were then recorded and summarized at 4, 12, 24, and 48 weeks after the KD therapy. The AEDs maintained unchanged from the first 12 weeks of the diet and then adjusted accordingly. The KD effect of seizure control was evaluated by Engel scale (12), including Grade I when completely seizure free, Grade II when seizures reduced by 90% to <100%, Grade III when seizures reduced by 50% to <90%, Grade IV when invalid (seizure reduction <50%). In our study, the KD treatment was considered effective when seizure reduction ≥50% (Grade I+II+III) compared to the frequency at baseline.

In the DS patients who received KD therapy for more than 12 weeks, Video Electroencephalogram (VEEG) was recommended for evaluation purpose. The background rhythm and interictal epileptic discharges were compared before and after the KD treatment. Cognition, language, and motor function of the patients were assessed according to their parents' or legal guardians' description.

### Statistical Analysis

SPSS 23.0 software was used for establishing the database. Chi-square test was used for statistical analysis to evaluate the efficacy of the KD treatment for the 60 patients at 12 weeks, according to variables including seizure onset age, the starting age of the KD treatment, the *SCN1A* mutation and the numbers of concurrent AEDs. *p* < 0.05 was regarded as statistically significant.

## Results

Among the 60 DS patients (34 boys and 26 girls). Fifty patients (83.3%) were detected with *SCN1A* mutations. The median seizure onset age was 5.5 months (3–11 months). Two or more seizure types were monitored during the course, including hemi-clonic seizures, generalized tonic-clonic seizures, focal seizures, myoclonic seizures, and atypical absence seizures. Fifty-seven patients had episodes of status epilepticus (seizure lasting for 30 min or longer). The seizures were fever-sensitive in all 60 patients. Twenty-seven patients had seizure episodes when taking a hot-water shower. twenty-Three patients had a post-vaccination seizure. The median starting age of KD therapy was 46 months old (14 months to 11 years and 6 months old). The duration of the KD ranged from 12 weeks to 54 months, with a median duration of 44 weeks. 41 (68.3%) patients remained on the diet for more than 24 weeks, 22 (36.7%) patients for more than 48 weeks. Nine of 60 patients, without significant improvement, discontinued the KD therapy from 12 weeks to 24 weeks after the initiation.

The seizures in 35 patients (35/60, 58.3%) were reduced over 50% at 12 weeks, including 20.0% (12/60) in Grade I, 20.0% (12/60) in Grade II, 18.3% (11/60) in Grade III, respectively, according to Engel scale (Table 1). Among them, the KD effect was observed within 2 weeks in 30 patients (30/35, 85.7%). At 24 weeks, 61.1% (25/41) of the patients had a >50% seizure reduction. At 48 weeks, 77.3% (17/22) had an over 50% reduction in their seizure frequency (Table 1). According to the statistical analysis, the efficacy of KD in DS patients did not correlate with the seizure onset age, the starting age of the KD treatment, the *SCN1A* mutation and the numbers of antiepileptic drugs combined with KD treatment (Table 2). With the treatment of KD in the 60 DS patients, 10 patients had ever been seizure free for 12 months to 24 months (The median duration was 20 months). Three patients withdrew 1 or 2 AEDs after being seizure free for 2 years since the initiation of the KD therapy.

**Table 1:** The efficacy of KD treatment in DS patients.

	**4 weeks** **(*n =* 60)**	**12 weeks** **(*n =* 60)**	**24 weeks** **(*n =* 41)**	**48 weeks** **(*n =* 22)**
Grade I	17 (28.3%)	12 (20.0%)	11 (26.8%)	5 (22.7%)
Grade II	7 (11.7%)	12 (20.0%)	9 (22.0%)	8 (36.4%)
Grade III	13 (21.7%)	11 (18.3%)	5 (12.2%)	4 (18.2%)
Grade IV	23 (38.3%)	25 (41.7%)	16 (39.0%)	5 (22.7%)
Effective (I-III)	37 (61.7%)	35 (58.3%)	25 (61.0%)	17 (77.3%)

*Grade I when completely seizure free, Grade II when seizures reduced by 90% ~ <100%, Grade III when seizures reduced by 50% ~ <90%, Grade IV when invalid (seizure reduction <50%). Effective when seizure reduction ≥50% (Grade I+II+III) compared to the frequency at baseline*.

**Table 2:** The analysis of the efficacy of KD therapy at 12 weeks in DS patients.

**Factors**	**Effective cases (%)**	**Ineffective cases (%)**	P^*^-value
Onset age			0.753
<6 months	21 (35.0)	16 (26.7)	
≥6 months	14 (23.3)	9 (15.0)	
The initiation age of KD			0.681
<3 years old	13 (21.7)	8 (13.3)	
≥3 years old	22 (36.7)	17 (28.3)	
*SCN1A* mutations			0.907
Positive	29 (48.3)	21 (35.0)	
Negative	6 (10.0)	4 (6.7)	
Numbers of AEDs			0.497
2	15 (25.1)	7 (11.6)	
3	13 (21.7)	12 (20.0)	
4	7 (11.6)	6 (10.0)	

* *p < 0.05 was regarded as statistically significant*.

The efficacy of 9 KD-effective patients declined after the treatment for 4~96 weeks. Two of the 5 patients with Grade I at 4 weeks dropped to Grade II at 8 weeks, one dropped to Grade III, two patients declined to Grade II and Grade III at 12 weeks and 24 weeks. Two of the 4 patients with Grade II at 12 weeks reduced to a lower rank with Grade III and Grade IV at 24 weeks, respectively. The remaining 2 patients declined to Grade IV at 48 weeks and 96 weeks.

In 10 KD-effective patients, the background rhythm of their EEG showed obvious improvement, and interictal epileptic discharges decreased significantly. The VEEG results of 3 patients with Grade I or Grade II were normal after 12 months with KD treatment. According to the description of their parents at the 24-week visits, cognitive function of 22 patients was improved including 4 patients with Grade III and 2 with Grade IV after 6 to 12 months with KD treatment. Language ability progressed in 14 patients, including 2 patients with Grade III. Motor function was improved in 13 patients including one patient with Grade III.

The main side effects of KD in the treatment process were gastrointestinal symptoms and metabolic disorders. Twenty-five patients had 1 to 3 kinds of side effects, including 6 with hypoglycemia, 4 with acidosis, 17 with constipation, 4 with diarrhea, 12 with weight stagnation or loss, 3 with urinary stones, 2 with abnormal liver function, and 4 with hyperlipidemia. Constipation, diarrhea, liver dysfunction and urinary stones were tolerable after adjustment of the KD and symptomatic treatment. One patient quit the KD therapy after the blood lipids were 10 times higher than normal.

## Discussion

Nowadays, despite the introduction of more than a dozen AEDs in the past 2 decades, about one third of patients with epilepsy remain refractory (2). The KD is an effective treatment for drug-resistant epilepsy in addition to treatments, such as vagal nerve stimulation or surgery therapy (5–7). A study of 61 children with refractory epilepsy treated with KD showed that the effective rates were 60.0, 57.0, 54.3, and 50.0% at 3, 6, 12, and 24 months after KD treatment (13). In patients with developmental and epileptic encephalopathy with genetic etiology, KD is more effective in patients with *SCN1A, KCNQ2, STXBP1*, and *SCN2A* mutations rather than patients with *CDKL5* mutations (7). This dietary protocol has also been validated as effective at stopping seizures in DS patients (8, 12–16). Nabbout et al. observed that 10 (66.6%) of 15 children with DS had a 75% reduction in seizures after 1 month of KD treatment. After 3 and 6 months of KD treatment, the effective rate decreased to 53.3%. Twelve months after KD treatment, the effective rate reduced to 33.3% (15). Among the studies investigating KD treatment in DS, our study enrolled the largest number of cases. A total of 60 DS patients were treated with KD. After 12 weeks of treatment, 35 children (35/60) had a >50% reduction in seizures, and the effective rate was 58.3%. At 24 weeks and 48 weeks, the effective rate was 61.1% (25/41) and 77.3% (17/22), respectively. The KD effect was observed within 2 weeks in most of the patients. This study further supported the efficacy of KD treatment in DS patents. It also showed a rapid response in most effective patients. However, the efficacy of some DS patients declined after the treatment for 4~96 weeks. Other studies also showed that the efficacy of KD decreased with the prolongation of KD treatment to patients with intractable epilepsy (15, 17). The specific mechanism of this phenomenon was unclear. A decrease of efficacy by KD treatment in some patients might be related to a loss of compliance, with difficult fine-tuning of the diet to maintain high ketone body levels (11). In DS patients, it was presumed that decreased expression or structural change of sodium channel subunits might also play a role, similar to the effect of some AEDs known as the honeymoon effect.

Multicenter studies have shown that there is no significant relationship between the efficacy of KD and the age of KD treatment. Than et al. showed that sex, the age at seizure onset, the starting age of KD treatment, numbers of AEDs combined with KD treatment in children with epilepsy had no significant relationship with the effect (18). A prospective study of 299 children with intractable epilepsy treated by KD showed that there was no significant difference in the efficacy of KD at 12 months between different age groups, different etiological groups (cryptogenic, symptomatic, primary epilepsy) and different types of epilepsy (19). However, some studies have shown that children aged over 10 years old are less effective than those aged <10 years old at 3 months of KD treatment, but this difference is not significant at 6 months and 12 months of KD treatment (20). In our study, the results showed that there was no significant relationship between the efficacy of KD treatment at 12 weeks and the age of seizure onset, the age of KD treatment, *SCN1A* mutation and AEDs. In our study, the results might be related to the small number of cases and the relatively small age range of KD treatment. Bough et al. explored KD treatment in 305 Sprague-Dawley rats for 22–126 days. It was found that the effect of KD treatment was age-dependent (21). That is, the younger the patients when the KD treatment started, the more significant the anti-epileptic effect of KD was. Kossoff et al. proposed that KD therapy should be considered earlier as an option for treatment of intractable epilepsy, because of its proven efficacy and the possible reduction of further AEDs combination (22). Another study recently showed that KD was highly effective and well-tolerated in infants with epilepsy (23). DS patients may consider KD therapy as an early treatment option.

KD treatment not only demonstrates good clinical efficacy for seizure control, but also significantly reduces the frequency of interictal epileptic discharges and improves the EEG background rhythm (24, 25). The longer with the treatment of KD, the more significant the improvement of the EEG background rhythm (26). A study investigated the effect of KD on the EEG features of 31 children with drug therapy-resistant epilepsy. At 1 week, a reduction in the interictal epileptic discharge of >50% was observed in 5 of the 31 cases; this increased to 14 cases at 1 month and 16 cases at 3 months (5). Improvement of EEG could also be seen in patients with a poor clinical KD efficacy (without significant reduction of seizure frequency) (26, 27). It was considered that EEG changes result from favorable electrical physiological effects of KD. This indicated that KD had a positive impact on the central nervous system and cortical neurons (27, 28). In our study, 10 DS patients showed obvious improvement in EEG, 3 of them had a normal finding after 12 months of treatment with KD. Our results were comparable to those of the previous reports. The EEG should be performed regularly and the numbers of DS patients with KD treatment should be expanded to further study the EEG changes.

Another positive effect of KD has been observed in children with drug-resistant epilepsy. It helped to improve the cognitive development and behavioral disorders, including hyperactivity, attention problems and autistic behavior (26). The improvement might be associated with the reduction of seizures and the numbers of AEDs combined. The behavioral improvement may be evident even in patients with a poor clinical KD efficacy (28). It is hypothesized that individuals with autistic behavior may have deficient glucose oxidation, and therefore, use ketone bodies as an alternative energy fuel in the brain (29). A logical explanation is difficult to establish because the mechanism of action of the KD is still not well-comprehended. KD affects some essential biochemical pathways that influence the function of the central nervous system (30). The increase of ketone bodies keeps the γ-aminobutyric acid (GABA) at a higher level, which may explain its beneficial effect on seizure control and behavior problems (31–33). In our study, cognitive function of 22 patients was improved, including 2 patients with Grade IV. Language ability progressed in 14 patients. Motor function was improved in 13 patients. Our findings support that KD improves cognitive function and behavioral impairment in DS patients. The limitation of our present study is that the mental-physiological development was assessed according to their parents' or legal guardians' description.

Ketogenic diet is a rigid diet rich in lipids and low in protein and carbohydrates in order to produce ketosis and simulates a starvation state. The side effects include hypoglycemia, metabolic acidosis, dehydration, lethargy, and gastrointestinal symptoms. The long-term adverse effects include weight loss, hyperlipidemia and nephrolithiasis. It is essential to know how to recognize the symptoms of some acute side effects, such as hypoglycemia and metabolic acidosis (22, 34). Therefore, the patients' weight, height, blood lipid and renal ultrasound should be monitored regularly during the KD treatment. In this study, the side effects of KD treatment were mainly gastrointestinal symptoms and metabolic disorders such as hyperlipidemia. Most of the side effects were tolerable after adjustment of the KD and symptomatic treatment.

A multidisciplinary team including pediatric neurologists and nutritionists should be established to resolve possible doubts and discuss side effects. Each patient may have an individually designed diet. It is essential to inform the patient and the family about the efficacy and side effects related to the KD, as well as the training of preparation of the KD food. The websites, videos and support groups may help in this education. In this study, 60 DS patients were followed up regularly by pediatric neurologists and nutritionists. It was helpful to solve the problems encountering during the KD therapy and reduce the abandonment of the diet.

## Conclusions

In summary, KD is an effective and safe treatment alternative in DS patients. The treatment of KD in DS has many advantages, including working rapidly, effective in more than half of the DS patients and tolerable adverse reactions. Therefore, KD therapy is recommended as an early option for drug resistant DS patients.

## Data Availability

All datasets generated for this study are included in the manuscript and/or the supplementary files.

## Author Contributions

XT provided clinical care to the patients and wrote the initial manuscript. JC, JZ, and XY participated in the follow up of the patients and prepared the tables. TJ, YaZ, and YW monitored the patients in hospital and carried out the statistical analysis. XY collected the literature. FF, XW, and YuZ reviewed and edited the manuscript and approved the final version.

### Conflict of Interest Statement

The authors declare that the research was conducted in the absence of any commercial or financial relationships that could be construed as a potential conflict of interest.
